# Epidemiology and Characteristics of Gastric Carcinoma in Childhood—An Analysis of Data from Population-Based and Clinical Cancer Registries

**DOI:** 10.3390/cancers15010317

**Published:** 2023-01-03

**Authors:** Michael Abele, Lisa Grabner, Tabea Blessing, Andreas Block, Abbas Agaimy, Christian Kratz, Thorsten Simon, Gabriele Calaminus, Sabine Heine, Selim Corbacioglu, Holger Christiansen, Dominik T. Schneider, Ines B. Brecht

**Affiliations:** 1 Pediatric Hematology/Oncology, Department of Pediatrics, University Hospital Tuebingen, 72076 Tuebingen, Germany; 2 Clinic of Pediatrics, Klinikum Dortmund, University Witten/Herdecke, 44137 Dortmund, Germany; 3 Department of Hematology and Oncology, University Medical Center Hamburg-Eppendorf, 20251 Hamburg, Germany; 4 Institute of Pathology, University Hospital Erlangen, Friedrich-Alexander-University Erlangen-Nuernberg, 91054 Erlangen, Germany; 5 Pediatric Hematology and Oncology, Hannover Medical School, 30625 Hannover, Germany; 6 Department of Pediatric Oncology and Hematology, University Children’s Hospital of Cologne, 50937 Cologne, Germany; 7 Department of Pediatric Hematology and Oncology, University Hospital Bonn, 53127 Bonn, Germany; 8 Pediatric Oncology and Hematology, Children’s Hospital Medical Center, University Clinics Homburg, 66421 Homburg, Germany; 9 Department of Pediatric Hematology, Oncology and Stem Cell Transplantation, University of Regensburg, 93053 Regensburg, Germany; 10 Department of Pediatric Oncology and Hematology, University of Leipzig, 04103 Leipzig, Germany

**Keywords:** gastric cancer, rare tumors, pediatric oncology, epidemiology

## Abstract

**Simple Summary:**

While gastric carcinoma is prevalent in adults and is one of the most common causes of cancer-related death, the disease is exceptionally rare in children. The aim of this analysis was to present peculiarities of gastric carcinoma in children and adolescents. A combination of *Helicobacter pylori* infection and tumor predisposition and/or immunodeficiency appears to contribute significantly to the risk of developing gastric carcinoma at a young age. In adolescents, these tumors are often diagnosed at an advanced stage of disease, which is associated with a poor prognosis and emphasizes the need for the development of new therapeutic strategies, such as molecular-targeted treatments.

**Abstract:**

(1) Background: Gastric carcinoma is an exceptionally rare tumor in childhood. Little is known about the etiology, epidemiology, and clinical features of pediatric gastric carcinomas. This analysis aimed to fill this gap by increasing knowledge about the occurrence of gastric carcinoma in childhood. (2) Material and methods: Data from gastric carcinoma cases diagnosed between 2000 and 2017/2018 were retrieved from the Surveillance, Epidemiology, and End Results Program (SEER) and the German Center for Cancer Registry Data. Data from patients <20 years of age were analyzed for patient- and tumor-related characteristics. In addition, clinical data from patients with gastric carcinoma registered in the German Registry for Rare Pediatric Tumors (STEP) were analyzed for diagnostics, therapy, and outcome. (3) Results: Ninety-one cases of gastric carcinoma, mainly in adolescents, were identified in the epidemiologic cancer registries. Among patients with recorded staging data, advanced tumor stages were common (66.7%). Within the follow-up period covered, 63.7% of patients with clinical follow-up data died. Eight pediatric patients with gastric carcinoma were enrolled in the STEP registry, among whom two were patients with hereditary *CDH1* mutations and another was a patient with Peutz–Jeghers syndrome. Three patients were found to have distinctly decreased immunoglobulin concentrations. All four patients in whom complete resection was achieved remained in remission. Three of the other four patients died despite multimodal therapy. (4) Conclusions: A combination of *Helicobacter pylori* infection and tumor predisposition and/or immunodeficiency appears to promote the development of gastric carcinoma in childhood. While patients with localized disease stages have a good chance of achieving durable remission through complete resection, patients with stage IV carcinomas face a dismal prognosis, highlighting the need to develop new strategies such as mutation-guided treatments.

## 1. Introduction

Gastric carcinoma in adults is a common disease, accounting for 5.6% of all cancers and 7.7% of all cancer-related deaths worldwide [1]. It is a multifactorial disease, and the main factors influencing carcinogenesis are *Helicobacter pylori* (*HP*), exogenous factors such as diet, tobacco smoking, and salt consumption, and genetic predisposition [2,3,4]. The vast majority of gastric carcinomas are histologically adenocarcinomas [2]. A distinction is made between adenocarcinomas arising from the cardia and from the more distal areas of the stomach, as they differ in both etiology and clinical course [5]. Since the early stages of the disease are often without clinical symptoms, the diagnosis is often made at an advanced stage of disease, resulting in an unfavorable prognosis with an average 5-year survival rate of approximately 40% [6]. In sharp contrast, gastric carcinomas in childhood are classified as very rare cancers, with a crude annual incidence rate <2/1.000.000, corresponding to roughly 0.1% of all pediatric cancers [7]. Only a few such cases have been clinically characterized in publications [8,9,10,11,12,13,14]. Consequently, the epidemiology, etiology, clinical characteristics, and treatment of pediatric gastric carcinomas, along with potential differences from adult characteristics, are poorly understood [8,14]. The rare occurrence of these tumors poses a serious challenge for treating physicians. The present analysis aims to improve our understanding of the epidemiology of gastric carcinomas in childhood and to characterize pediatric patients with gastric carcinomas by presenting clinical features, management, and outcomes.

## 2. Materials and Methods

We retrieved data from the German Center for Cancer Registry Data (ZfKD). This national cancer registry receives data from the population-based cancer registries of all of the German federal states, as required by the Federal Cancer Registry Data Act [15]. For comparison, we also retrieved data from the Surveillance, Epidemiology, and End Results Program (SEER) of the National Cancer Institute, which collects and publishes data from population-based cancer registries covering nearly 50% of the US population (SEER Research Plus Data, 18 Registries, Nov 2020 Submission) [16]. The retrieved datasets consisted of all patients recorded by the ZfKD and SEER who met the following inclusion criteria: diagnosis of malignancy with the International Classification of Diseases (ICD) codes C16.0–C16.9; initial diagnosis under the age of 20 years and between 2000 and 2017 (ZfKD) or 2018 (SEER). Patients with gastric carcinoma were identified by filtering based on the recorded International Classification of Diseases for Oncology (ICD-O) histology codes and included in this analysis. Importantly, due to structural changes in the reporting requirements, not all federal registries reported to the ZfKD over the entire period. The ZfKD only received data from all federal registries from 2009 to 2015. An overview of the respective reportings by year and registry, including reported follow-ups, can be found in Supplemental Material Table S1.

Pseudonymized data for patient- and tumor-related features (age at diagnosis, cancer site, and histology per ICD-O, status per TNM-Classification of Malignant Tumors (TNM), grading, recorded treatment, and outcome) were included in the obtained datasets from the ZfKD and SEER. A limitation of the present study is that TNM staging information was missing in approximately 50% of cases in the ZfKD records. In the results section, frequencies are related to cases with recorded data.

We calculated the crude incidence rates for the under-20 age group as the number of cases per person-years, calculated according to the average population count between 2009 and 2015, as reports from all state registries were obtained from the ZfKD during this period. Data on the childhood population were obtained from the Federal Statistical Office of Germany [17]. 

Further analysis was based on patients registered in the database of the German Registry for Rare Pediatric Tumors (STEP), which is a prospective clinical registry of the German Society for Pediatric Oncology and Hematology (GPOH). The inclusion criteria were diagnosis with a gastric carcinoma and an age under 18 years at diagnosis. Pseudonymized data for patient-, tumor-, and treatment-related characteristics were collected based on original source data using standardized case report forms from the STEP registry. Staging was based on the TNM classification by the American Joint Committee on Cancer (AJCC; Supplemental Material Table S2) [18]. All patients and/or their guardians gave informed consent for data collection and analysis at diagnosis. The STEP registry was approved by the institutional review boards of both the University Erlangen (Re. No. 4340) and the participating hospitals.

## 3. Results

We identified 91 cases of gastric carcinoma in children and adolescents in the epidemiologic cancer registries (ZfKD: *n* = 31; SEER: *n* = 60). The characteristics of these patients are shown in Table 1. Most patients were diagnosed in adolescence, and 80.2% were 15–19 years old at diagnosis. The median age at diagnosis for patients recorded by the ZfKD was 17.6 years (range: 0–19 years). In both the ZfKD and SEER data, a slight female preponderance was observed (57.1%). Almost all gastric carcinomas were adenocarcinomas, with poorly cohesive adenocarcinomas, namely signet ring cell carcinomas, being the most commonly specified histologic subtype (38.5%). Based on the Laurén classification, diffuse type tumors were therefore most frequent, followed by intestinal-type and indeterminate-type carcinomas [19]. However, in 47.5% of cases, the ICD-O morphology code 8140/3 (adenocarcinoma, not otherwise specified) was reported, so no further histologic assignment was possible in these patients. There were no cases of Epstein-Barr virus (EBV)-associated lymphoepithelioma-like gastric carcinoma. Tumors were mainly located in the cardia (*n* = 19), body (*n* = 12), and antrum (*n* = 13) of the stomach and only rarely in the curvature region. In terms of TNM staging, the T stage was relatively evenly distributed between T1 and T4. Lymph node metastases were detected in 51.2% of cases. Distant metastases were found in 61.1% of cases. Accordingly, advanced disease stages, especially stage IV, were common (66.7%). Tumors were also frequently poorly differentiated on pathologic examination. Most gastric carcinomas were classified as high-grade tumors (86.9%). While most of these observations were identical for ZfKD and SEER, certain differences were noted. In the SEER cases, more tumors were categorized as overlapping lesions, and we found a higher frequency of distant metastases and stage IV cancers (ZfKD: 56.3%, SEER: 71.4%). The ZfKD also had a slightly higher proportion of patients younger than 15 years of age (ZfKD: 25.8%, SEER: 16.7%). 

While the ZfKD-recorded treatment modalities were limited, more detailed information on therapy was available from the SEER registry (Table 2). Chemotherapy was the primary treatment modality (71.6% overall), whereas the majority of patients recorded by the SEER registry did not receive resection of the primary tumor (62.1%). In contrast, 52.9% of patients with gastric carcinoma recorded by the ZfKD underwent surgery. Radiotherapy was performed infrequently (13.7% overall) and was most commonly used as adjuvant therapy. Within the recorded follow-up period, 63.7% of patients with gastric carcinoma died (Table 3). While the ZfKD recorded 48.4% of patients as deceased (77.8% in stage IV), the rate of deceased patients in the SEER registry was 71.7%. According to the SEER data, the median time to last follow-up was 8 months (mean: 26.7 months, range 0–168 months). Consistently, the median time from diagnosis to death based on the ZfKD data was 10.2 months (range 0–28 months). The median age at death in these patients was 18.8 years (range 13.5–20.9 years). 

Using cases recorded by the ZfKD from 2009 to 2015, we were able to calculate a crude incidence rate for gastric carcinoma in children and adolescents in Germany of 0.16 per million during this period.

Eight patients with gastric carcinoma were enrolled in the STEP registry, with a median age at diagnosis of 16.1 years (range 12.7–17.4 years). The characteristics of these patients are shown in Table 4. The most common symptoms that led to diagnostic testing were abdominal pain (*n* = 4), stool irregularity (*n* = 2), and anemia (*n* = 2). These symptoms were usually present between one and three months prior to diagnosis. The initial clinical diagnosis was made by gastroscopy (*n* = 7) or imaging (*n* = 1) and subsequently confirmed by histopathological analysis. All carcinomas were classified as adenocarcinomas, with signet ring cell carcinoma being the most common subtype (*n* = 3). Based on the Laurén classification, diffuse type (*n* = 3) and intestinal type (*n* = 2) carcinomas were present, as well as mixed type tumors (*n* = 3) [19]. Signet ring cell segments were detected in all three mixed type tumors. Precancerous lesions were intestinal metaplasia in the setting of chronic gastritis (*n* = 2) and hyperplastic polyps with intestinal metaplasia (*n* = 1). One of the two patients with hereditary diffuse gastric cancer (HDGC) was found to have moderate foveolar hyperplasia consistent with in situ carcinoma. EBV was not detected in any tissue sample. Overexpression of HER2/neu and microsatellite instability were not found in all samples examined (*n* = 6; *n* = 4). Peutz–Jeghers syndrome was detected in one patient during a subsequent diagnostic workup after the diagnosis of gastric carcinoma. Two patients were diagnosed with a germline *CDH1* mutation causing HDGC. In one of these patients, gastric carcinoma was detected at an early stage during a cancer screening gastroscopy. Immunoglobulin concentrations of IgA and IgG were significantly decreased at diagnosis in three patients. Two of these patients were diagnosed with type A gastritis. One patient already had a known autosomal recessive agammaglobulinemia. On histopathologic examination, seven patients showed evidence of chronic gastritis. Infection with *HP* was detected in gastric tissue samples in six patients. Advanced disease stages were frequently observed at diagnosis (5× stage IV). The peritoneum was the most common site of metastases. All patients in whom complete resection (CR) was achieved by gastrectomy were still in remission at their last follow-up (*n* = 4). In the other four cases, resection of the primary tumor was not reasonably feasible due to advanced metastatic spread. Three of these patients died within a short time, and the fourth patient showed progression on therapy at the last follow-up. The first-line chemotherapies included 5-fluorouracil, cisplatin/oxaliplatin, and docetaxel (*n* = 5). In addition to chemotherapy, immunotherapies and regional deep hyperthermia were also administered.

## 4. Discussion

Gastric carcinomas predominantly arise in older adulthood, with less than 10% of all gastric cancers occurring in patients younger than 45 years of age, although an increase in incidence has recently been noted in young adults [20,21]. These tumors are very rare in childhood, accounting for less than 0.1% of all gastric carcinomas [22]. In our analysis of patients recorded in the ZfKD and SEER registries as well as the STEP registry, we found an increase in gastric carcinoma in adolescence, which has also been confirmed in other reports [8,14]. However, young children may also be affected. In contrast to gastric carcinoma in adults, where a preponderance of males was detected, we found a preponderance of females among pediatric patients with gastric carcinoma [23]. The reason for the female predominance in childhood gastric cancer remains unclear. As in adulthood, almost all gastric carcinomas are adenocarcinomas [2]. Although there are different classifications regarding the distinct histopathological subtypes (World Health Organization, Laurén, Japanese Gastric Cancer Association), these are not relevant with regard to therapy in the adult treatment guidelines [19,23,24]. Signet ring cell feature is a characteristic attribute of some gastric adenocarcinomas, and there is controversy about its possible influence on prognosis [10,25,26]. While signet ring cell carcinomas account for only 20% of adult gastric carcinomas, we found a much higher frequency of 38.5% for this histological subtype [22]. While non-cardia gastric cancer is the most common site of gastric carcinoma worldwide (80%), associated with *HP* infection, salt intake, and alcohol consumption, proximal gastric cancer is more common in adults in North America and Western Europe and is linked with obesity and gastroesophageal reflux [23,27]. Although we observed the gastric cardia as a common site of pediatric gastric carcinoma, the majority of gastric cancers were located at other sites. Accordingly, we found a high frequency of *HP* positivity in pediatric gastric carcinomas (6/8 STEP patients). Although this characteristic is not recorded in the epidemiologic cancer registries and thus could not be verified in the larger cohorts, *HP* has been detected in many other published pediatric gastric cancers [8,14]. Because *HP* is a proven risk factor for adult gastric carcinoma, and carcinogenesis involves a cascade from chronic inflammation to atrophy and sometimes intestinal metaplasia, this suggests that *HP* is also an important risk factor for the development of gastric cancer in childhood [3,11,23,28,29]. *HP* infections in childhood are common, and the prevalence of *HP* infections in children varies from 10% in the US and Western Europe to as high as 80% in India, Africa, or Latin America [30]. Notably, the frequency of *HP* positivity in our patients was distinctly higher than expected in the German population. In a Turkish cohort of 750 pediatric patients undergoing upper gastrointestinal endoscopy, 52% were *HP* positive. Of these, 74% had chronic gastritis and 6.2% and 2.8% had already developed atrophy and intestinal metaplasia, respectively, but no cases of cancer were found [30]. This indicates that *HP* infection alone may not be sufficient to trigger carcinogenesis in childhood. While, in adulthood, the duration of chronic gastritis and atrophy contribute to the risk of cancer development, as do lifestyle and diet, these risk factors do not appear to play a comparably important role in carcinogenesis in childhood [2,31]. In children, a genetic predisposition to cancer is often more relevant to carcinogenesis than in adulthood [32]. For gastric carcinomas, a number of cancer predisposition syndromes are known in adulthood, such as HDGC, gastric adenocarcinoma and proximal polyposis of the stomach (GAPPS), and familial intestinal gastric cancer (FIGC) [4]. In addition, there are cancer predisposition syndromes that are not unique to but are also associated with gastric carcinomas, such as Li-Fraumeni syndrome, Lynch syndrome, familial adenomatous polyposis, juvenile polyposis, and Peutz–Jeghers syndrome [4,33,34,35,36]. While truly hereditary cases account for approximately 3% of adult gastric cancers, accumulation within families occurs in up to 10% of cases [4]. HDGC is the most common cancer predisposition for gastric carcinoma and is caused by germline variants in *CDH1* and *CTNNA1* [37]. It is inherited in an autosomal dominant manner with high penetrance and increases the lifetime risk of developing gastric cancer to approximately 80% (in addition to a 60% lifetime risk of developing breast cancer in women) [10,37]. In the STEP cohort, HDGC was found in two of the eight patients, and Peutz–Jeghers syndrome was found in another patient. Thus, cancer predisposition syndromes were more common than in adults. In addition to one case of autosomal recessive agammaglobulinemia, the immunoglobulin concentrations of IgA and IgG were markedly decreased at diagnosis in two other patients. Several immunodeficiencies, such as X-linked and autosomal recessive agammaglobulinemia, common variable immunodeficiency, and IPEX syndrome have already been associated with an increased risk of developing gastric carcinoma in adulthood and, in some cases, in pediatric case reports [38,39,40,41,42,43]. Based on our findings, immunodeficiencies may also play an important role in the development of gastric cancer in childhood. Taken together, our results suggest that a combination of *HP* infection and tumor predisposition and/or immunodeficiency contributes significantly to the risk of developing gastric carcinoma at a young age.

Because symptoms of gastric carcinoma are usually nonspecific, a correct diagnosis is often delayed by several months, which has also been acknowledged in other reports [10,14]. This aspect and frequent poor differentiation contribute to advanced disease stages at diagnosis. We found a markedly higher incidence of stage IV carcinomas (66.7% vs. 41%) and poor differentiation (86.9% vs. 65%) than in adults [22]. While stage IV carcinomas are associated with a poor prognosis, complete resection of the primary tumor at a localized stage of disease provides a good chance for durable remission in adults [23]. This also seems to be true for gastric carcinoma in childhood, as all STEP patients with CR remained in remission, whereas the patients with stage IV cancer recorded by the ZfKD had a survival rate of only 22% (adults 5–15%) [44,45]. The observed difference in the frequency of deceased patients between the ZfKD and SEER registry is presumably also related to this aspect, as the SEER registry recorded more patients with stage IV cancers and a lower proportion of patients with resection of the primary tumor. The treatment regimens applied, including first-line chemotherapies, were adopted from adult therapy protocols (Table 5) [23]. However, in the most recent treatment recommendations, taxane-based triplet chemotherapy is not recommended for advanced disease stages because no clear survival benefit over doublet regimens was observed, and it has a higher toxicity [23]. While adult therapy guidelines recommend the addition of trastuzumab in HER2-positive advanced gastric cancer (prevalence 10–20%), none of the tumor samples studied in the STEP cohort (*n* = 6) had HER2/neu overexpression [23,46]. PD-L1 status was rarely assessed at diagnosis, and only one of the STEP patients was treated with PD-L1 inhibition, and only then in the fourth line of therapy. Notably, while PD-L1 inhibition with nivolumab is recommended in adults with advanced cancers with a combined positive score ≥ 5 regardless of *HP* status, it has been reported that *HP*-positive patients had a higher risk of non-response to PD-L1 inhibition [23,47,48]. Because *HP* positivity was common in pediatric gastric carcinomas, this could affect the efficacy of this treatment. Currently, the treatment of gastric cancer in childhood should be based primarily on the evidence-based adult guidelines and should be undertaken in close collaboration with adult oncologists, particularly with regard to the treatment of microsatellite unstable cancers, which are associated with excellent long-term outcomes after PD-L1 inhibition [23,36]. The evaluation at diagnosis should include genetic testing for cancer predisposition (Table 6) in addition to analysis of *HP*, HER2/neu, and PD-L1 status. Children and adolescents with a family history of gastric carcinoma, as well as those with already diagnosed cancer predisposition syndromes and immunodeficiencies associated with gastric cancer, should undergo regular endoscopic surveillance. This can detect gastric carcinoma at a localized stage of disease, as in one of our patients, leading to a significant improvement in survival. Furthermore, in these patients with cancer predisposition, *HP* eradication treatment should be considered in cases of confirmed chronic gastritis with detection of *HP* to reduce the risk of gastric cancer [49]. In addition, consistent recording of these cancers in clinical cancer registries for rare childhood tumors, such as the STEP registry, is essential to successively improve the management of gastric carcinomas in childhood through the continuous recording and evaluation of diagnostic and therapeutic modalities.

## 5. Conclusions

Our analysis provides a detailed picture of the characteristics of gastric carcinoma in childhood. Complete resection at a localized stage of disease provides a good chance of durable remission, whereas patients with stage IV carcinomas face a dismal prognosis. In contrast to carcinogenesis in adults, tumor predisposition syndromes and immunodeficiencies are much more common in childhood and, together with *HP* infections, appear to promote early cancer development. Close collaboration with adult oncologists is needed to provide optimal treatment of pediatric patients with gastric carcinoma, in consideration of these characteristics of childhood gastric carcinoma and a greater impact of long-term therapy-associated morbidity [51]. Therefore, an interdisciplinary network of cancer experts such as the European Cooperative Study Group for Pediatric Rare Tumors (EXPeRT) is essential to ensure optimal treatment of these tumors in childhood in accordance with the latest research findings and clinical experience [52].

## Figures and Tables

**Table 1:** Patient characteristics and clinical features of gastric carcinoma in patients aged 0–19 years registered at the German Center for Cancer Registry Data 2000–2017 and in the Surveillance, Epidemiology, and End Results Program 2000–2018. Proportions are calculated based on cases with recorded data. Abbreviation: n.a., not applicable.

Characteristics	German Center for Cancer Registry Data (ZfKD) 2000–2017 (*n* = 31)		Surveillance, Epidemiology, and End Results Program (SEER) 2000–2018 (*n* = 60)	
	Count	Proportion	Count	Proportion
Sex				
Male	13	41.9%	26	43.3%
Female	18	58.1%	34	56.7%
Age at diagnosis				
<10	5	16.1%	0	0%
10–14	3	9.7%	10	16.7%
15–19	23	74.2%	50	83.3%
Histology				
Adenocarcinoma	30	96.8%	60	100.0%
Signet cell carcinoma thereof	10	32.3%	25	41.7%
Squamous cell carcinoma	1	3.2%	0	0%
Localization				
Cardia (C16.0)	9	29.0%	10	16.7%
Fundus (C16.1)	0	0%	3	5.0%
Stomach body (C16.2)	8	25.8%	4	6.7%
Antrum (C16.3)	4	12.9%	9	15.0%
Pylorus (C16.4)	1	3.2%	4	6.7%
Lesser curvature (C16.5)	0	0%	1	1.7%
Greater curvature (C16.6)	0	0%	3	5.0%
Overlapping lesion (C16.8)	0	0%	6	10.0%
Not specified (C16.9)	9	29.0%	20	33.3%
T stage				
1	4	30.8%	11	44.0%
2	2	15.4%	7	28.0%
3	3	23.1%	3	12.0%
4	4	30.8%	4	16.0%
X/no data	18	n.a.	35	n.a.
N stage				
0	5	41.7%	16	51.6%
1	4	33.3%	10	32.3%
2	2	16.7%	2	6.5%
3	1	8.3%	3	9.7%
X/no data	19	n.a.	29	n.a.
M stage				
0	7	43.8%	14	36.8%
1	9	56.3%	24	63.2%
X/no data	15	n.a.	22	n.a.
Disease stage				
I	5	31.3%	8	22.9%
II	2	12.5%	1	2.9%
III	0	0%	1	2.9%
IV	9	56.3%	25	71.4%
Unknown/no data	15	n.a.	25	n.a.
Grading				
Low-grade (I/II)	4	20.0%	4	9.8%
High-grade (III)	16	80.0%	37	90.2%
Unknown/no data	11	n.a.	19	n.a.

**Table 2:** Treatment characteristics of gastric carcinoma in patients aged 0–19 years registered in the Surveillance, Epidemiology, and End Results Program 2000–2018.

Treatment Modalities	Count	Proportion
Surgery of primary tumor		
Local excision	1	1.7%
Partial gastrectomy	8	13.3%
Near total/total gastrectomy	12	20.0%
Surgery not otherwise specified	1	1.7%
No resection of primary tumor	36	60.0%
Unknown	2	3.3%
Lymph node surgery		
Resection of ≥4 regional lymph nodes	17	28.3%
No lymph node resection	31	51.7%
Unknown	12	20.0%
Radiation		
Yes	8	13.3%
neoadjuvant	2	3.3%
adjuvant	6	10.0%
No	52	86.7%
Chemotherapy		
Yes	45	75.0%
No/unknown	15	25.0%

**Table 3:** Outcome of/last reported follow-up for gastric carcinoma in patients aged 0–19 years registered at the German Center for Cancer Registry Data 2000–2017 and in the Surveillance, Epidemiology, and End Results Program 2000–2018.

Status	German Center for Cancer Registry Data (ZfKD) 2000–2017 (*n* = 31)		Surveillance, Epidemiology, and End Results Program (SEER) 2000–2018 (*n* = 60)	
	Count	Proportion	Count	Proportion
Alive	16	51.6%	17	28.3%
Deceased	15	48.4%	43	71.7%

**Table 4:** Characteristics of pediatric patients with gastric carcinoma registered with the German Registry for Rare Pediatric Tumors (STEP). Abbreviations: *H.p.*, *Helicobacter pylori*; 5-FU, Fluorouracil; FU, Follow-up. * Chemotherapy: Carboplatin, Ifosfamide, Etoposide/Adriamycin.

Gender	Age at Diagnosis (Years)	TNM	Stage/Grading	Site of Metastases	Pre-Existing Diseases	Surgery of Primary Tumor	Therapy of Metastases	Medical Therapy	Status at Last FU	Time Since Diagnosis (Years)
f	14.3	T1aN0M0	IA/G3	-	Chronic gastritis (*H.p.* pos.), *CDH1* mutation	Gastrectomy	-	-	Alive off therapy	2.8
f	16.1	T1aN0M0	IA/G3	-	Chronic type A gastritis (*H.p.* neg.), IgG/IgA low	Resection (endoscopic)	-	-	Alive off therapy	0.7
f	16.1	T3N0M0	IIA/G3	-	Chronic gastritis (*H.p.* pos.)	Gastrectomy	-	5-FU, Leucovorin, Oxaliplatin, Docetaxel (FLOT, 6 cycles, 4 thereof neoadjuvant)	Alive off therapy	1.8
f	16.0	TxN + M1	IV/G3	Ovaries, Peritoneal carcinomatosis, lymph nodes	Chronic gastritis (*H.p.* pos.), IgG/IgA low, *CDH1* mutation	-	Resection of an ovarian metastasis	5-FU, Leucovorin, Oxaliplatin, Docetaxel (FLOT, 5 cycles)	Dead of disease	1.2
m	15.4	T3N0M1	IV/G2	Singular peritoneal metastasis	Autosomal recessive agammaglobulinemia, Chronic type A gastritis (*H.p.* pos.)	Gastrectomy	Resection	-	Alive off therapy	6.5
f	12.7	T4N1M1	IV/G2/3	Peritoneal carcinomatosis	Dysembryoplastic neuroepithelial tumor (WHO I°), Peutz–Jeghers syndrome	-	-	5-FU, Cisplatin, Docetaxel/Epirubicin (6 cycles), 5-FU, Leucovorin, Irinotecan, Oxaliplatin (FOLFOXIRI, 3 cycles), regional deep hyperthermia + chemotherapy (2×) *	Dead of disease	0.8
f	16.7	TxN3M1	IV/G3	Lung, liver, lymph nodes, bones, bone marrow	Chronic gastritis (*H.p.* pos.)	-	-	5-FU, Leucovorin, Oxaliplatin, Docetaxel (FLOT, 6 cycles), 5-FU, Leucovorin, Irinotecan (FOLFIRI, 13 cycles), Ramucirumab/Paclitaxel, Nivolumab	Dead of disease	1.0
f	17.4	T4N2M1	IV/G3	Peritoneal carcinomatosis	Chronic gastritis (*H.p.* pos.)	-	-	5-FU, Cisplatin, Docetaxel (6 cycles)	Alive on therapy	0.3

**Table 5:** Treatment algorithm for gastric carcinoma, simplified illustration, based on the guidelines of the European Society for Medical Oncology [23] (Staging: as shown in Supplemental Material Table S2).

Stage	Surgery	Further Therapy
IA	Endoscopic or surgical resection	-
IB—III	Radical gastrectomy + D2 lymphadenectomy	Perioperative chemotherapy as a triplet chemotherapy regimen including a fluoropyrimidine, a platinum compound, and docetaxel (standard: 5-fluorouracil, leucovorin, oxaliplatin, docetaxel (FLOT)) Adjuvant chemotherapy, if no preoperative therapy has been performed Radiotherapy might be considered after resection with microscopic residual tumor (R1) Microsatellite instable cancer: no adjuvant chemotherapy, neoadjuvant downstaging with FLOT possible
IV—or locally advanced unresectable disease	IV: only in highly selected cases	Doublet chemotherapy regimen including a fluoropyrimidine and a platinum compound ○ Plus additional trastuzumab in HER2-positive tumors ○ Plus additional nivolumab if PD-L1 combined positive score ≥ 5 (pembrolizumab possible if PD-L1 combined positive score ≥ 10) Second line therapy: Ramucirumab (VEGFR inhibitor) + paclitaxel

**Table 6:** Overview of cancer predisposition syndromes associated with gastric carcinoma and the respective underlying genetic alterations [20,23,50].

Genetic Alteration	Associated Cancer Predisposition Syndrome
*CDH1*, *CTNNA1*	Hereditary Diffuse Gastric Cancer (HDGC)
*APC* promotor 1B	Gastric Adenocarcinoma and Proximal Polyposis of the Stomach (GAPPS)
*APC*	Familial Adenomatous Polyposis (FAP)
*BMPR1A*, *SMAD4*	Juvenile polyposis syndrome
*TP53*	Li-Fraumeni syndrome
*MLH1*, *MSH2*, *MSH6*, *PMS2*	Lynch syndrome
*STK11* (*LKB1*)	Peutz–Jeghers syndrome

## Data Availability

The data that support the findings of this study are available on request from the corresponding author. The data are not publicly available due to privacy restrictions.

## Supplementary Materials

The following supporting information can be downloaded at: https://www.mdpi.com/article/10.3390/cancers15010317/s1, Table S1: ZfKD—reports and follow-up received by state registries; Table S2: TNM staging for gastric carcinoma according to the American Joint Committee on Cancer (AJCC), 8th edition.
