# Supplementary material for: Epidemiology and Characteristics of Gastric Carcinoma in Childhood—An Analysis of Data from Population-Based and Clinical Cancer Registries
Source: Cancers (Basel). 2023 Jan 3;15(1):317. doi: 10.3390/cancers15010317 (PMC9818931; doi:10.3390/cancers15010317)
Supplement: Supplementary file 1 [file cancers-15-00317-s001.zip › cancers-2089099-supplementary.pdf]

Table S1. ZfKD – reports and follow-up received by state registries.

State registry		Active reporting	Reported Follow-up until
Schleswig-Holstein		2000 – 2017	31.12.2017
Hamburg		2000 – 2017	31.12.2017
Lower Saxony		2003 – 2017	31.12.2017
Bremen		2000 – 2017	31.12.2017
North Rhine-Westphalia		2006 – 2017	31.12.2017
Subregistries	*Dusseldorf*	2006 – 2017	31.12.2017
	Cologne	2006 – 2017	31.12.2017
	Münster	2000 – 2017	31.12.2017
	Detmold	2006 – 2017	31.12.2017
	Arnsberg	2006 – 2017	31.12.2017
Hesse		2007 – 2016	31.12.2016
Rhineland-Palatinate		2000 – 2016	31.12.2016
Baden-Württemberg		2009 – 2017	31.12.2017
Bavaria		2002 - 2017	31.12.2017
Saarland		2000 – 2017	31.12.2017
Berlin		2000 – 2015	31.12.2015
Brandenburg		2000 – 2015	31.12.2015
Mecklenburg-Western Pomerania		2000 – 2015	31.12.2015
Saxony		2000 – 2015	31.12.2015
Saxony-Anhalt		2000 – 2015	31.12.2015
Thuringia		2000 – 2015	31.12.2015

Table S2. TNM staging for gastric carcinoma according to the American Joint Committee on Cancer (AJCC), 8th edition [18].

Stage	T	N	M
IA	T1	N0	M0
IB	T1	N1	M0
	T2	N0	M0
IIA	T1	N2	M0
	T2	N1	M0
	T3	N0	M0
IIB	T1	N3a	M0
	T2	N2	M0
	T3	N1	M0
	T4a	N0	M0
IIIA	T2	N3a	M0
	T3, 4a	N2	M0
	T4a	N1	M0
	T4b	N0	M0
IIIB	T1, 2	N3b	M0
	T3, 4a	N3a	M0
	T4b	N1, 2	M0
IIIC	T3, 4a, 4b	N3b	M0
	T4b	N3a	M0
IV	any T	any N	M1
